# The Perceived Corporate Social Responsibility of Major Sport Organizations by the German Public: An Empirical Analysis During the COVID-19 Pandemic

**DOI:** 10.3389/fspor.2021.679772

**Published:** 2021-09-16

**Authors:** Tim F. Thormann, Pamela Wicker

**Affiliations:** Department of Sports Science, Bielefeld University, Bielefeld, Germany

**Keywords:** corporate social responsibility, crisis, football, Olympic Games, sport governing body, professional sports

## Abstract

Sport governing bodies have played a special role in society during the first wave of the COVID-19 pandemic. Following stakeholder theory and consumption capital theory, this study investigated the actions of the German Bundesliga (DFL), Union of European Football Associations (UEFA), and the International Olympic Committee (IOC) during this phase as perceived by the German population and through the lens of corporate social responsibility (CSR). Based on a representative sample of the German resident population (*N* = 1,000), the study examined the individual characteristics that influenced the perceived CSR of these organizations and what population clusters emerged from this perception. The survey applied a CSR scale that was previously validated in a professional team sports context. The results confirmed the equally strong applicability of the scale to the sport governing context. Cluster analysis yielded three distinctive clusters, namely, “supporters,” “neutral observers,” and “critics.” Regression analyses and the cluster analysis identified those with frequent consumption and high involvement in sport as rating the actions of the three sport organizations more positively. They are also more strongly represented in the “supporters” cluster. In contrast, those threatened the most by the virus are overrepresented in the “critics” cluster.

## Introduction

The worldwide COVID-19 pandemic has influenced public life and the world of sport in a way that has never existed before. Since this pandemic and the resulting lockdowns can be considered an unforeseen external shock to the sport system, sport organizations have constantly faced trade-offs when deciding how to proceed with competitions and events. On the one hand, organizations need revenue from sport competitions and events, which are their core business, and they also have an economic responsibility to their partners and suppliers (Preuss, 2005). Most parties have higher chances to survive if sport events and competitions take place, which is a reality that has resulted in debatable decisions such as the relocation of international matches to foreign countries due to travel restrictions as can be seen in the UEFA Champions League (UEFA, 2021). For example, matches between German and English teams in round 16 were relocated to Budapest (Hungary) to circumvent the existing travel restrictions between Germany and the UK (UEFA, 2021).

On the other hand, the role of these sports bodies in society suggests that they should behave in a societally responsible manner (Reiche, 2014). One of the terms often used by sport officials during the pandemic is the societal responsibility of the sport [DFL, 2020; DOSB, 2020]. This term is anchored on policy documents, outlining that sport represents a central component of social life, which connects people with different cultural backgrounds, conveys societally important values, and fosters public debate and inclusion (DOSB, 2016). The benefits of sports in addressing socio-economic challenges in society and its elite athletes fulfilling a role model function have been recognized by policymakers. These two contributions to society are core arguments for the funding of elite sports and sports for all programs (DOSB, 2016; Council of the European Union, 2017). Due to the trade-off between economic viability and societal responsibility, a question arises concerning the extent to which sport organizations were able to demonstrate societal responsibility during the ongoing COVID-19 pandemic. At the time this study was conducted, the first wave of the pandemic had finished, restrictions were loosened, and multiple future waves could not be anticipated. Hence, the perceptions regarding the actions of the organizations concentrated on the first months of the global pandemic (March–June 2020).

Initial market research indicated that the actions of some of these sport organizations were received critically by the German population. For example, people wondered why high-paid players were allowed to return to action while contact and travel restrictions were still in place for the majority of the German population (Nielsen Sports, 2020; Westdeutscher Rundfunk, 2020), ultimately questioning the societal responsibility of sport organizations. While business-related travels were allowed for some industries in Germany such as the automobile and information technology (IT) industry, contact restrictions prohibited professionals in the service sector, such as barbers and the hospitality industry, to practice their professions, indicating an exemption of contact restrictions for professional sport athletes. German politicians also criticized the irresponsible behavior of football organizations and questioned the special role that European football held during the pandemic (Deutschlandfunk, 2021).

Societal responsibility is usually investigated under the concept of corporate social responsibility (CSR) (e.g., Smith and Westerbeek, 2007; Babiak and Wolfe, 2009). The German Football League (DFL) is legally constituted as a corporation, to which the principles of CSR apply in the same way they do to corporations in other industries. As legally constituted non-profit organizations that abide by the laws in their registered countries, sport governing bodies such as the Union of European Football Association (UEFA) and the International Olympic Committee (IOC) are also subject to CSR principles (Chelladurai, 2016). Previous studies provided several examples of CSR activities of non-profit sport organizations (Chelladurai, 2016), the findings of which highlighted the importance for non-profit organizations to implement CSR activities to project a positive image, receive public subsidies, and enhance the goodwill of their core stakeholders (Chelladurai, 2016). The COVID-19 pandemic represents a special context for sport organizations to demonstrate CSR, since CSR activities during the pandemic do not cover specific community programs but are rather concerned with showing general responsibility beyond the economic obligations of an organization, i.e., being a role model for social distancing, giving back to those in need, or fulfilling the legal COVID-19 rules.

The purpose of this study was to investigate the CSR of sport governing bodies during the first wave of the COVID-19 pandemic as perceived by the German population. The German population was investigated, because German Olympic athletes are partly funded through tax-payer money in Germany (Wicker et al., 2021), and such money is also used to cover the security costs during match days of the German Bundesliga, UEFA Champions League, and UEFA Europa League. In return, the German population might expect social responsibility from the three organizations to legitimatize the spending of tax-payer money. Therefore, this study sought to answer the following research questions: (1) Does the employed CSR scale demonstrate adequate reliability and validity when applied to sport governing bodies? (2) What individual characteristics influence the perceived CSR of these organizations? (3) And what population clusters emerge according to CSR perception?

Based on the stakeholder theory proposed by Freeman (1984), sport organizations rely on strong acceptance from their largest external stakeholders, such as the population, so that they can claim exceptional rules for themselves in times of crises. Freeman (1984) defined a stakeholder as “any group or individual who can affect or is affected by the achievement of the organization's objective” (p. 6). Contrary to the investigation of other external stakeholders in sport (e.g., fans and sponsors), who are often investigated qualitatively, it is difficult to assemble the perception of the population through interviews, which leads to a quantitative assessment. The heterogeneity and large size of the population make a quantitative assessment advantageous. Therefore, a representative online survey among the German population was conducted using a previously validated CSR scale, which was developed in the professional team sport context in Iran with a focus on fans (Montazeri et al., 2017). Hence, this study applied this scale to sport governing bodies in the Western context. The DFL, UEFA, and IOC were selected for two reasons. First, the decisions of all three organizations affect the participation of German teams and athletes in international and national competitions. While the DFL only decides about national competitions, the UEFA and IOC decide about the operations of major international competitions, such as the 2020 European Championships and the 2020 Olympic Games. Second, Google search results for March to June 2020 indicate that information about their actions was frequently made available to the German population through newspaper articles, TV news, and talk shows, the availability of which is an important prerequisite to evaluate their actions (Du et al., 2010).

By answering the presented research questions, this study enhances understanding of the perception of the population of CSR activities as a core stakeholder of sport. Furthermore, the application of the CSR scale in a sport governing context provides a starting point for future quantitative assessments of CSR perceptions by different stakeholders. Especially in critical times, it is crucial for sport organizations to individually foster socially responsible behavior and communication (Inoue et al., 2017; Babiak and Kihl, 2018; Walzel et al., 2018).

## Theoretical Framework and Literature Review

### Conceptualization of CSR

Many conceptualizations of CSR draw from the stakeholder theory proposed by Freeman (1984). This theory recognizes that organizations are responsible for and should create value for a variety of stakeholders instead of only focusing on wealth creation for the sake of shareholders (Freeman, 1984). Accordingly, several scholars highlighted the responsibility organizations have to their consumers, employees, governments, future generations, the natural environment, and society as a whole (Turker, 2009; Pérez and Rodríguez del Bosque, 2013b). Furthermore, pressure from external stakeholders is one of the main reasons for sport organizations to adopt CSR practices, which underlines the importance of the stakeholder perspective in the CSR construct (Babiak and Wolfe, 2009; Kolyperas et al., 2015).

This theoretical foundation fits the seminal conceptualization of CSR of Carroll (1979, 1991), which is multidimensional in nature and encompasses the following four dimensions: economic, legal, ethical, and discretionary responsibilities. The economic responsibility includes the obligation of an organization to produce valuable products for consumers at a good price, while being profitable and creating wealth for shareholders. The legal dimension refers to the responsibility of an organization to obey the law and respect governmental regulations in operations. Ethical responsibility goes beyond the normative expectations of society and implies morally right behavior and meeting ethical norms even though they are not always bound by law. Organizations gain legitimacy if they follow the expectations society has for ethically right behavior. Lastly, discretionary responsibility includes voluntary philanthropic or altruistic behavior, targeting the well-being and development of society (Carroll, 1979, 1991).

The multidimensionality of the CSR conceptualization by Carroll is also reflected in the definition of the European Commission (2002). “Corporate social responsibility is about companies having responsibilities and taking actions beyond their legal obligations and economic or business aims. These wider responsibilities cover a range of areas but are frequently summed up as social and environmental where social means society broadly defined, rather than simply social policy issues” (n.p.). The present conceptualization considers the multidimensionality of CSR.

### Consumption Capital and CSR Perceptions

Consumption capital might influence CSR perception in the general population. Generally speaking, consumption capital theory states that individuals gain knowledge about a good or entity through repetitive consumption, which, in turn, increases the utility of consumption (Stigler and Becker, 1977). The theory implies that imperfect information exists about the consumption of the goods; in this case, the actions of the three sport organizations (Stigler and Becker, 1977). Accordingly, repetitive consumption of sport leads to better knowledge about players, teams, and organizations (e.g., Wicker et al., 2012), so that their activities can be better evaluated. Since the general public usually does not proactively seek information about the socially responsible behavior of sports organizations, this information must be provided by the media. For example, TV shows and news reports about the activities of sport organizations (Du et al., 2010), and especially the actions of the three selected organizations, were covered. Accordingly, higher levels of consumption capital were expected to influence CSR perceptions in this study.

### CSR in Sport: Empirical Evidence

Several studies have been conducted examining the CSR of sport organizations in various contexts, including professional team sports (Walzel et al., 2018), major sport events (e.g., Babiak and Wolfe, 2006), sport leagues (e.g., Reiche, 2014), and sport federations (e.g., Filizöz and Fişne, 2011; Walters and Tacon, 2011). The next paragraphs review the types of implemented CSR activities, the drivers of these activities, and the employed research methods (Table 1).

**Table 1:** Summary of researched corporate social responsibility (CSR) activities, drivers, and the employed research methods.

**Findings**	**References**
**CSR activities**	
Community programs with a focus on sport participation, youth education, and development	Babiak and Kihl, 2018; Rowe et al., 2019
Corporate giving for charitable causes, including donations, ticket giveaways, and fundraising campaigns	Lacey and Kennett-Hensel, 2016; Inoue et al., 2017
Voluntary actions that represent ethical and discretionary activities	Babiak and Wolfe, 2006
**CSR drivers**	
External pressure from other stakeholders, including fans, communities, media, and competing clubs as well as internal resources represent main CSR drivers	Babiak and Wolfe, 2009; Kolyperas et al., 2015
Financial autonomy and human resources represent drivers for sport federations	Zeimers et al., 2020
Lack of financial and human resources represent the strongest constraints for implementing formal CSR strategies	Walters and Tacon, 2011
Role model function, economic motives, and political pressure drive CSR activities for the DFL	Reiche, 2014
**Methods**	
Qualitative interviews	Douvis et al., 2015; Babiak and Kihl, 2018
Qualitative observations, focus groups, and content analysis	Walters and Tacon, 2011; Banda and Gultresa, 2015
Qualitative case studies	Filizöz and Fişne, 2011; Reiche, 2014; Rowe et al., 2019
Mixed methods	Blumrodt et al., 2013; Davies and Moyo, 2017
Quantitative survey with three-to five item CSR scale	Chang et al., 2016; Lacey and Kennett-Hensel, 2016; Inoue et al., 2017

Concerning CSR activities, previous research interest was on activities within the community (e.g., “NBA Cares”), with a focus on sport participation, youth education, and development (e.g., Babiak and Kihl, 2018; Rowe et al., 2019). Other activities such as donations, ticket giveaways, and fundraising campaigns can be categorized as corporate giving for charitable causes (e.g., Lacey and Kennett-Hensel, 2016; Inoue et al., 2017). This concentration on the instrumental citizenship perspective that views CSR as a functional tool to achieve organizational goals is surprising, as the first study by Babiak and Wolfe (2006) chose to investigate the ethical and discretionary dimensions of CSR fulfilled by voluntary, implicit actions. Since then, the ethical and discretionary dimensions of CSR have rarely been investigated (Walzel et al., 2018).

Several drivers that foster CSR activities of organizations were studied, especially external pressure from other stakeholders including competing clubs and the internal resources that drive the CSR activities of professional sport teams (Babiak and Wolfe, 2009; Kolyperas et al., 2015). The drivers for sport federations include financial autonomy and human resources (Zeimers et al., 2020). A lack of these resources also represents the strongest constraints for implementing formal CSR strategies (Walters and Tacon, 2011). For the DFL, their role model function, economic motives, and political incentives influenced CSR activities (Reiche, 2014). All three organizations currently under investigation constantly interact with external stakeholders, especially during the pandemic. Additionally, they possess financial autonomy and human resources; thus, they are supposed to show societally responsible behavior, which is important to legitimize their special role during crises.

Scholars have investigated CSR in sports using different methods. Most studies applied qualitative research paradigms (e.g., Babiak and Trendafilova, 2011; Douvis et al., 2015). Especially in professional team sports, qualitative approaches were preferred (Walzel et al., 2018), with data being collected *via* interviews (e.g., Douvis et al., 2015; Babiak and Kihl, 2018) or observations, focus groups, and content analyses (e.g., Walters and Tacon, 2011; Banda and Gultresa, 2015). Most qualitative studies were conducted as case studies of different teams (e.g., Davies and Moyo, 2017; Babiak and Kihl, 2018), leagues (Reiche, 2014; Banda and Gultresa, 2015), and federations (Filizöz and Fişne, 2011). Few researchers used a mixed-method approach to answer their research questions, specifically by conducting interviews and surveys (Babiak and Trendafilova, 2011; Blumrodt et al., 2013; Davies and Moyo, 2017). Exclusive quantitative research was deployed by using surveys (e.g., Walters and Tacon, 2011; Chang et al., 2016; Lacey and Kennett-Hensel, 2016). However, most of the questionnaires used only short three- to five-item scales for measuring CSR or CSR perception, which barely covered the multidimensional nature of CSR. An exception is the study by Walters and Tacon (2011), who quantitatively measured CSR while respecting the multidimensional measure of the concept. Consequently, a lack of quantitative, empirical measurement of CSR activities was constantly mentioned as a drawback of the existing research.

While existing research has yielded valuable insights, a few shortcomings can be observed. First, previous studies put a strong emphasis on the implementing organizations, which are mostly professional sport teams. Second, the focus was on the possible positive outcomes of CSR activities for the organizations themselves, instead of directly analyzing and understanding the main stakeholder, i.e., society itself. This omission is surprising as the CSR concept includes the idea of responsibility to society as outlined above. Both shortcomings were addressed in the present study, as it focused on three sport-governing bodies (DFL, UEFA, and IOC) and how their activities during the pandemic were perceived based on different population characteristics. Third, the strong focus on qualitative research in the form of case studies led to a call for a more quantitative evaluation of CSR actions (Walters and Tacon, 2011; Douvis et al., 2015; Walzel et al., 2018). Fourth, as the perception stakeholders have of CSR activities are crucial for mutually beneficial relationships (Walters and Tacon, 2011; Douvis et al., 2015; Walzel et al., 2018), it is important to survey them about their perceptions.

### Determinants of CSR Perception

Although stakeholder-oriented CSR actions of sport organizations are crucial for their success, little is known about the individual characteristics that influence CSR perception in the sports context. For this purpose, general management literature was consulted, starting with the socially conscious consumer, and followed by the perceptions of the CSR actions of organizations outside of sports (Table 2). Hence, the present study bridged the gap between CSR research in sports and general management (Walzel et al., 2018).

**Table 2:** Summary of determinants of CSR perception.

**Determinant**	**Findings**	**References**
Gender	Socially conscious consumers tend to be female	Berkowitz and Lutterman, 1968; Gupta and Singh, 2017
Gender	Women are more likely to perceive CSR activities as positive	Patino et al., 2014; Kim and Kim, 2016
Age	Socially conscious consumers tend to be young	Anderson and Cunningham, 1972; Gupta and Singh, 2017
Age	Inconsistent age effects in CSR perception	Tian et al., 2011; Mueller, 2014
Education	Socially conscious consumers are highly educated	Berkowitz and Lutterman, 1968; Roberts, 1995
Education	Higher education positively influences CSR perception	Youn and Kim, 2008; Lee, 2019
Income	Socially conscious consumer has high or at least average income	Webster, 1975; Gupta and Singh, 2017
Income	Moderate and high-income groups more committed to CSR activities	Youn and Kim, 2008; Patino et al., 2014
Consumption capital	Consumption capital influences the evaluation of organizational activities	Du et al., 2010; Wicker et al., 2012
Involvement	Involvement influences the CSR perception positively	Assael, 1992; McGehee et al., 2003

The characteristics of socially conscious consumers have been widely studied, with a special interest in socio-demographic characteristics (Anderson and Cunningham, 1972; Webster, 1975). Starting with gender, findings were fairly consistent, in that the socially responsible consumer tended to be female (e.g., Berkowitz and Lutterman, 1968; Webster, 1975; Gupta and Singh, 2017). Most CSR studies confirmed these results by showing that women were more likely to perceive CSR actions as positive (e.g., Patino et al., 2014; Kim and Kim, 2016), while only a few studies detected no gender effect on perceived CSR (e.g., Mueller, 2014).

Turning to age, existing research has shown that the socially oriented consumer tends to be young, reflecting a negative correlation between age and social orientation (e.g., Berkowitz and Lutterman, 1968; Anderson and Cunningham, 1972; Gupta and Singh, 2017). For the perception of CSR activities, the findings are not consistent. While some scholars suggest that middle-aged consumers react positively to CSR activities (e.g., Carrigan et al., 2004; Tian et al., 2011), others detected no age differences (Mueller, 2014) or consistent effects (Pérez and Rodríguez del Bosque, 2013a).

Regarding education, existing research confirmed that the socially conscious consumer is highly educated (e.g., Berkowitz and Lutterman, 1968; Roberts, 1995) and has a good job (Anderson and Cunningham, 1972). Accordingly, higher education was found to have a positive influence on perceived CSR in most studies (e.g., Youn and Kim, 2008; Lee, 2019), while only a few studies showed that educational level had no effect (e.g., Pérez and Rodríguez del Bosque, 2013a).

Concerning income, previous research found that the socially conscious consumer has high (Berkowitz and Lutterman, 1968; Webster, 1975) or at least average income (Gupta and Singh, 2017). Furthermore, results suggested a high level of satisfaction with the income level for socially conscious consumers (Gupta and Singh, 2017). Moderate- (Tian et al., 2011) and high-income groups were also more favorable and more committed to the CSR activities of organizations (Youn and Kim, 2008; Patino et al., 2014).

Other factors like involvement might also influence the perception of CSR activities. Involvement is referred to “as an unobservable state of motivation, arousal, or interest that is evoked by a particular stimulus or situation and has drive properties” (Havitz et al., 1994, p. 39). It is characterized by being enduring and continuous and assesses facets like pleasure and value within decision-making (McGehee et al., 2003). Accordingly, it is often used to explain the consumer behavior and decision-making process of an individual (Assael, 1992). Involvement seems important for the perception of CSR activities, since consumers with different levels of involvement process information differently. Specifically, consumers with a high level of involvement proactively seek information, analyze it, and form an evaluation based on the analyses. On the contrary, consumers with a low level of involvement process information rather passively, which leads to a low evaluation of brands or their actions (Assael, 1992; McGehee et al., 2003). In the given context, involvement refers to the involvement of respondents with sport in general, which refers to the value and importance of being involved in sport on site or *via* television. Previously, involvement served as a positive mediator between CSR perceptions and behavioral outcomes such as attendance frequency (Inoue et al., 2017). Hence, it might affect the perception of CSR activities.

## Method

### Research Context

To classify the measures of the three organizations, the pandemic context in Germany and the actions of the three sport organizations are to be presented in the next sections. On January 27th, the first COVID-19 case in Germany was reported by the German Federal Ministry of Health. Following several hundred additional cases within the next 6 weeks, the Federal Ministry of Health and the Federal Ministry of Interior recommended the cancelation of all events with more than 1,000 visitors, which led to the first “ghost game” in the history of the German Football Bundesliga. On the 12th of March 2020, the ministers of education and culture decided to close schools countrywide until the end of the Easter holidays in the middle of April. On the same day, the government decided that retailers, theaters, museums, and grassroots sport centers must be closed indefinitely. On March 17th, the minister of interior announced extensive travel restrictions for all countries within and outside the EU (Federal Ministry of Health, n.d.; Federal Ministry of Interior, Building, and Community, 2020).

In the world of sports, some sport organizations reacted earlier than others to protect fans and players in equal ways. However, most of them bowed to the pressure of external stakeholders. For example, the National Basketball Association (NBA) was the first sport league to suspend their ongoing season (March 13th) when two players of the Utah Jazz team tested positive for the virus (Aschburner, 2020). Shortly after, other sport organizations such as the DFL, the UEFA, and the IOC reacted to the external pressure exerted by fans, athletes, and especially politics. On March 13th, the DFL first announced that it would continue with the next Bundesliga match day as planned without spectators before politicians canceled the first games. Following the dynamic of the day, the DFL suspended their season at least until April 2nd and later until May 16th (Zeit, 2020). As a consequence of the Europe-wide suspension of the leagues, the UEFA decided on March 17th to postpone the 2020 European Championship to 2021 and the UEFA Champions League and Europa League to later in the year (ESPN, 2020). Lastly, the IOC postponed the Tokyo Olympic Games on March 24th until summer 2021 after the Canadian Olympic Committee announced to boycott the Games if they were held as scheduled, while thousands of athletes complained about plans to stage the event despite the pandemic (Futterman, 2020).

The three organizations insisted on the right of free practice, similar to other industries. Because of that, the governments allowed them to continue their competitions in compliance with hygiene rules (German Government, 2021). On May 16, 2020, the Bundesliga restarted as the first major league worldwide, while the UEFA held their Champions League tournament from August 12th to August 23rd in Porto. The IOC rescheduled the Tokyo 2020 Olympic Games to July 2021.

### Data Collection

This study is based on data from a representative online survey of the German resident population. The data collection was conducted with the support of the independent market research company, Toluna Germany. The data was gathered between 26 June and 6 July 2020 closely after the first German lockdown ended, using a random multistage sampling procedure. Minimum age of 18 was required to participate in the survey. The sample was designed to be representative of the German adult resident population with regards to gender, age, and federal state. The respective quotas were taken from the most recent German micro census 2017 (Federal Statistical Office, 2020). Participants who speeded or just clicked through the questionnaire were identified and excluded. Moreover, the answers of participants were checked for plausibility and validity. After data cleaning, a representative sample of *N* = 1,000 residents remained for the empirical analysis.

### Questionnaire and Variables

The questionnaire was designed by the research team and consisted of seven different sections, including questions about sport participation, interest, involvement, consumption capital, perceived CSR, well-being, and socio-demographic characteristics of the respondents. Table 3 provides an overview of the variables used in the analysis and their summary statistics. The introduction informed respondents that their participation was voluntary, anonymous, and the data were only collected and analyzed for scientific purposes.

**Table 3:** Overview of variables and summary statistics (*N* = 1,000).

**Variable**	**Description**	**Percentage**	**Mean**	**SD**
CSR	Additive CSR index of 17 items/17 (Table 5)	—	4.16	1.30
Weighted CSR	CSR scale weighted with the factor loadings from the CFA (Table 5)	—	3.93	1.23
CSR DFL	Additive CSR index of 17 items for the DFL/17	—	4.21	1.27
CSR UEFA	Additive CSR index of 17 items for the UEFA/17	—	4.23	1.34
CSR IOC	Additive CSR index of 17 items for the IOC/17	—	4.05	1.29
Sport participation	Weekly number of hours of active sport (average over the last 4 weeks)	—	7.37	7.92
Sport interest	General interest in sport (1 = no interest at all; 5 = very strong)	—	3.40	1.08
Involvement	Additive involvement index of 9 items (see Table 4; 1 = low involvement, 7=high involvement)/9	—	4.48	1.85
CC sport recent	Consumption of pandemic sport news in last 2 weeks (1 = less than once per week, 5 = more than once a day)	—	2.42	1.25
CC sport lockdown	Consumption of pandemic sport news in March and April 2020 (1 = less than once per week, 5 = more than once a day)	—	2.29	1.24
CC news recent	Consumption of pandemic news in last 2 weeks (1 = less than once per week, 5 = more than once a day)	—	3.50	1.22
CC news lockdown	Consumption of pandemic news in March and April 2020 (1 = less than once per week, 5 = more than once a day)	—	3.77	1.16
CC interest DFL	Level of interest in the German Bundesliga (1 = no interest at all, 5 = very strong)	—	3.12	1.36
CC frequency DFL	Frequency of German Bundesliga games watched (1 = never, 5 = always)	—	2.92	1.29
CC interest UEFA	Level of interest in UEFA competitions (1 = no interest at all, 5 = very strong)	—	3.11	1.32
CC frequency UEFA	Frequency of UEFA competitions watched (1 = never, 5 = always)	—	3.06	1.26
CC interest IOC	Level of interest in Olympic Games (1 = no interest at all, 5 = very strong)	—	3.19	1.25
CC frequency IOC	Frequency of Olympic Games competitions watched (1 = never, 5 = always)	—	3.16	1.22
WHO-5	Additive well-being index of five items (see Table 7; 0 = very low well-being, 100 = very high well-being)	—	55.94	24.30
Male	Gender of respondent (1 = male, 0 = female)	49.3	—	—
Age	Age (in years)	—	49.62	16.03
Age squared	Age*Age	—	2718.22	1540.39
Low education	Highest educational level is below A-levels (1 = yes, 0 = no)	45.3	—	—
A-levels	University entry degree (i.e., A-levels; 1 = yes, 0 = no)	25.1	—	—
University	University or university of applied sciences degree (1 = yes, 0 = no)	29.6	—	—
Income	Personal monthly net income (1 = up to 500€, 9 = over 4,000€)	—	4.88	2.19
Worker	Full-time or part-time employment (1 = yes, 0 = no)	60.4	—	—
Short-time work	Short-time work (1 = yes, 0 = no)	1.1	—	—
Student	Still in education (1 = yes, 0 = no)	4.8	—	—
Pensioner	Pensioner (1 = yes, 0 = no)	27.2	—	—
Unemployed	Unemployed (1 = yes, 0 = no)	6.5	—	—
Migrant	Respondent has a migration background; i.e., respondent or at least one parent has a non-German nationality (1 = yes, 0 = no)	14.3	—	—

*CC = consumption capital*.

The questionnaire started with assessing the participation and interest respondents have in sport. Respondents were asked to report their average number of weekly hours of sports for the last 4 weeks (*sports participation*) and their general interest in sports (*sports interest*) on a five-point scale. Involvement was measured with a validated involvement scale (McGehee et al., 2003) consisting of nine involvement items in a semantic differential format (Table 4). The Cronbach's alpha of the scale was 0.973, suggesting construct reliability (Hair et al., 2010). The final involvement variable (*involvement*) represented the average of the nine items. Pandemic-related consumption capital was assessed with four questions on a five-point scale, with respondents being asked to state how frequently they followed sport-specific pandemic news in the last two weeks (*CC sport recent*) and during the first German lockdown of the pandemic in March and April (*CC sport lockdown*). The same two questions followed for news regarding pandemic-specific news in the same time periods (*CC news recent; CC news lockdown*).

**Table 4:** Items included in the involvement index (ranging from 1 to 7).

**Item no**.	**1**		**7**	**Mean**
1	Unimportant	-	Important^a^	4.34
2	Boring	-	Interesting	4.68
3	Irrelevant	-	Relevant	4.49
4	Means nothing	-	Means a lot^a^	4.29
5	Worthless	-	Valuable^a^	4.40
6	Mundane	-	Fascinating	4.60
7	Unappealing	-	Appealing^a^	4.53
8	Uninvolving	-	Involving^a^	4.60
9	Not needed	-	Needed	4.34
	Involvement index (mean of all items)			4.48
	Cronbach's α			0.973

a *Item was reverse coded*.

Afterward, respondents were randomly assigned to one of the three organizations, resulting in similar-sized sub-samples for the DFL, UEFA, and IOC. This randomization was implemented to reduce the length and cognitive complexity of the survey. Hence, each respondent was only presented with sport-specific consumption capital questions and the CSR scale for one of the three organizations. For the DFL and IOC, the sport-specific consumption capital consisted of two questions asking for the general interest in the competition of the respective sport governing body (*CC interest*) and how often those competitions are watched (*CC frequency*). The corresponding competitions for the DFL and IOC are the first two divisions of the German Football Bundesliga and the Olympic Games, respectively. Since the UEFA would have organized two competitions in 2020, i.e., the European Championships and the rest of Champions League and Europa League, participants received four sport-specific consumption capital questions, two for each competition, and a mean value was created for the interest and frequency component.

At the heart of the survey was a CSR scale. Previous research has not agreed on a universally applicable scale (Decker, 2004) and existing scales take the industry specifics of CSR into account (e.g., Fatma et al., 2016; Alvarado-Herrera et al., 2017). This study applies the validated scale from Montazeri et al. (2017), which was specifically developed for the sport industry. It is based on the conceptualization of CSR by Carroll (1979, 1991) including the four dimensions of economic (*economic*, four items), philanthropic (*philanthropic*, five items), ethical (*ethical*, four items), and legal responsibility (*legal*, four items). The items were translated by three experts into German and adapted to fit the present research context of sport-governing bodies. The originally created scale comprised the environment as a separate dimension, which was less applicable to the COVID-19 context and was consequently deleted. To test its reliability and validity in the sport governing body context and answer research question 1, a second-order confirmatory factor analysis (CFA) was conducted (Table 5). One item in the economic dimension loaded weakly on the latent construct and was deleted. The model showed a good fit as the comparative fit index (CFI) was 0.964, even exceeding the recommended value of 0.9. Moreover, the root mean square error of approximation (RMSEA) was below the cut-off criterion of 0.08 with a value of 0.073, and the standardized root mean square (SRMR) was also below the cut-off criterion of 0.1 with a value of 0.0336 (Hair et al., 2010). Construct reliability was established for all latent factors as all composite reliabilities were above 0.8 and exceeded the recommended value of 0.6. In addition, all Cronbach's alphas exceeded the threshold of 0.7, indicating good reliability (Hair et al., 2010). The remaining manifest variables loaded significantly on their latent constructs, with all the loadings but one exceeding 0.7 (Jöreskog and Sörbom, 1986). The factor loadings for the four dimensions are were with values above 0.89 and they loaded significantly on the general factor CSR. The high factor loadings, in combination with the high correlation between the four dimensions and the CSR scale (Table 6), indicate good validity of the measured scale. As all values exceed the recommended thresholds, the scale as a whole and each dimension deemed good reliability and validity, supporting previous research (Montazeri et al., 2017). The final unweighted CSR measure (*CSR*) was the average of 17 items. Additionally, a weighted CSR measure (*Weighted CSR*) was calculated by multiplying the factor loadings of the second-order CFA with the respective mean values, then the four dimensions were summed up and divided by 4.

**Table 5:** Confirmatory factor analysis for the CSR scale.

**Latent construct**	**Indicator**	**λ**	**S.E**.	**SD**	**β**	**CR**	**Cronbach's α**
Economic					0.892^***^	0.825	0.805
	The X gives importance to spectators' satisfaction.	0.794^***^	0.127	1.746			
	The X tries to achieve long-term and sustained success.	0.824^***^	0.123	1.622			
	The X keeps a strict control over costs.	0.824^***^	0.125	1.648			
	The X endeavors to increase spectators.	0.467^a^	—	1.571			
Philanthropic					0.911^***^	0.931	0.934
	The X supports cultural and social events in the community.	0.850^***^	0.028	1.541			
	The X gives financial and non-financial support to NGOs	0.832^***^	0.028	1.518			
	The X supports activities related to health and wellness in the community.	0.886^***^	0.028	1.570			
	The X helps to solve social and ethical problems in the community.	0.854^***^	0.023	1.634			
	The X is committed to improve the welfare of the community.	0.845^a^	—	1.610			
Ethical					0.997^***^	0.917	0.916
	The X obeys the principle of fair play in the competition.	0.849^***^	0.027	1.674			
	The X obeys the ethical norm, which society requires.	0.876^***^	0.025	1.650			
	The X gives accountability to fans' criticism and demands.	0.830^***^	0.026	1.618			
	The X avoids unethical behavior.	0.874^a^	—	1.666			
Legal					0.967^***^	0.925	0.933
	The X tries to implement rules and regulations.	0.880^***^	0.026	1.612			
	The X respects the rights of fans beyond the legal requirements.	0.860^***^	0.027	1.588			
	The X respects rules and regulations defined by law.	0.868^***^	0.021	1.623			
	The X ensures that operations meet all legal standards.	0.865^a^	—	1.625			
	Cronbach's α of total scale						0.966

*Displayed are the standardized factor loading for the indicators (λ) and the latent constructs (β)*;

a *Reference category*;

*** *p < 0.001; CR, composite reliability*.

**Table 6:** Correlation matrix.

	**Item**	**1**	**2**	**3**	**4**	**5**	**6**	**7**	**8**	**9**	**10**	**11**	**12**	**13**	**14**	**15**
1	CSR	1														
2	Weighted CSR	1	1													
3	Economic	0.876	0.877	1												
4	Philanthropic	0.922	0.910	0.729	1											
5	Ethical	0.950	0.955	0.766	0.846	1										
6	Legal	0.930	0.937	0.768	0.780	0.883	1									
7	Sport participation	0.067	0.064	0.073	0.097	0.042	0.030	1								
8	Sport interest	0.193	0.191	0.186	0.193	0.160	0.169	0.340	1							
9	Involvement	0.307	0.305	0.275	0.303	0.276	0.272	0.123	0.540	1						
10	CC sport lockdown	0.227	0.244	0.193	0.250	0.189	0.198	0.092	0.391	0.551	1					
11	CC news recent	0.112	0.112	0.162	0.082	0.082	0.097	0.010	0.168	0.238	0.334	1				
12	CC news lockdown	0.093	0.095	0.145	0.049	0.062	0.102	−0.010	0.134	0.144	0.242	0.722	1			
13	CC frequency	0.339	0.338	0.316	0.315	0.300	0.316	0.126	0.488	0.739	0.530	0.231	0.156	1		
14	WHO-5	0.050	0.049	0.021	0.066	0.048	0.045	0.094	0.113	0.064	0.102	0.033	−0.002	0.083	1	
15	Male	−0.078	−0.080	−0.046	−0.053	−0.100	−0.087	−0.004	0.157	0.242	0.248	0.155	0.073	0.258	0.133	1
16	Age	−0.072	−0.071	−0.018	−0.087	−0.078	−0.073	−0.186	−0.181	0.036	−0.023	0.195	0.147	0.010	0.093	0.299
17	Age squared	−0.068	−0.067	−0.012	−0.085	−0.074	−0.069	−0.177	−0.173	0.027	−0.027	0.194	0.151	0.004	0.099	0.221
18	Low education	−0.009	−0.009	−0.014	−0.007	0.005	−0.019	−0.018	−0.166	−0.053	−0.099	−0.045	−0.039	−0.089	−0.066	−0.050
19	A-levels	0.027	0.026	−0.005	0.045	0.022	0.030	0.002	0.066	0.002	0.044	−0.035	−0.054	0.026	0.008	−0.077
20	University	−0.016	−0.015	0.020	−0.035	−0.027	−0.008	0.018	0.119	0.057	0.067	0.082	0.095	0.073	0.064	0.127
21	Income	0.013	0.011	0.018	0.036	0.002	−0.011	0.050	0.194	0.194	0.193	0.081	0.002	0.189	0.196	0.245
22	Workers	0.107	0.106	0.068	0.122	0.097	0.100	0.062	0.139	0.115	0.105	−0.068	−0.106	0.135	0.037	0.021
23	Short-time worker	−0.051	−0.052	−0.057	−0.031	−0.048	−0.055	0.068	−0.039	−0.068	−0.055	−0.051	−0.070	−0.053	−0.042	−0.066
24	Student	0.018	0.020	0.017	−0.004	0.030	0.028	0.004	0.046	−0.091	0.019	−0.016	0.044	−0.027	−0.048	−0.081
25	Pensioner	−0.084	−0.083	−0.049	−0.088	−0.085	−0.081	−0.085	−0.134	−0.046	−0.053	0.121	0.125	−0.066	0.070	0.094
26	Unemployed	−0.056	−0.054	−0.037	−0.067	−0.044	−0.052	−0.001	−0.057	−0.037	−0.107	−0.049	−0.025	−0.103	−0.139	−0.114
27	Migrant	0.048	0.047	0.024	0.055	0.049	0.044	0.068	0.038	−0.070	0.011	0.008	0.019	−0.008	−0.032	−0.026
28	DFL	0.024	0.024	0.012	0.025	0.011	0.040	0.013	0.045	0.031	0.004	−0.042	−0.043	−0.070	−0.027	−0.030
29	UEFA	0.036	0.035	0.028	0.042	0.045	0.014	0.030	−0.024	0.014	0.005	0.062	0.045	0.008	−0.004	0.046
30	IOC	−0.060	−0.059	−0.039	−0.068	−0.055	−0.055	−0.043	−0.021	−0.045	−0.009	−0.020	−0.002	0.062	0.031	−0.016
	**Item**	**16**	**17**	**18**	**19**	**20**	**21**	**22**	**23**	**24**	**25**	**26**	**27**	**28**	**29**	**30**
16	Age	1														
17	Age squared	0.988	1													
18	Low education	0.245	0.243	1												
19	A-levels	−0.291	−0.275	−0.527	1											
20	University	0.009	−0.004	−0.590	−0.375	1										
21	Income	0.058	0.022	−0.214	−0.049	0.280	1									
22	Workers	−0.356	−0.416	−0.183	0.091	0.113	0.334	1								
23	Short-time worker	−0.082	−0.086	0.039	−0.017	−0.026	−0.051	−0.130	1							
24	Student	−0.369	−0.312	−0.157	0.248	−0.064	−0.204	−0.277	−0.024	1						
25	Pensioner	0.628	0.673	0.220	−0.188	−0.062	−0.146	−0.755	−0.064	−0.137	1					
26	Unemployed	−0.071	−0.081	0.086	−0.050	−0.047	−0.202	−0.326	−0.028	−0.059	−0.161	1				
27	Migrant	−0.108	−0.111	−0.050	0.007	0.048	0.001	0.027	0.066	0.055	−0.076	0.008	1			
28	DFL	0.005	0.001	−0.063	0.022	0.048	0.031	0.034	0.027	−0.010	0.002	−0.074	−0.028	1		
29	UEFA	−0.015	−0.014	0.056	−0.037	−0.026	−0.004	−0.022	0.007	0.020	−0.036	0.089	0.020	−0.499	1	
30	IOC	0.010	0.013	0.007	0.015	−0.023	−0.027	−0.012	−0.034	−0.010	0.034	−0.015	0.007	−0.500	−0.500	1

*All correlation r > 0.063 are statistically significant (p < 0.05)*.

The levels of well-being of the respondents were captured, with the well-being index developed by the World Health Organization (WHO, 1998), which has already been applied in different fields (Topp et al., 2015), including sport (Schlegel et al., 2017). It consists of five items assessing how respondents felt during the German peak of the pandemic on a six-point scale (from 0 to 5; Table 7). The final index was obtained by adding up all variables and multiplying the raw score by 4, resulting in a final score between 0 and 100 (*WHO-5*). Cronbach's alpha was 0.919 suggesting construct reliability.

**Table 7:** Items included in the well-being index (WHO-5).

**No**.	**Item (0 = at no time; 5 = all of the time)**	**Mean**
	*Over the last four weeks…*	
1	I have felt cheerful and in good spirits	2.78
2	I have felt calm and relaxed	2.86
3	I have felt active and vigorous	2.65
4	I woke up feeling fresh and rested	2.76
5	My daily life has been filled with things that interest me	2.94
	WHO-5 index (additive index of all items multiplied by 4)	55.96
	Cronbach's α	0.919

The survey finished with an assessment of different socio-demographic characteristics, including gender (*male*), age (*age*), highest level of education (*low education*; *A-levels, university*), income (*income*), occupation (*worker, short-time work, student, pensioner, unemployed*), and migration background (*migrant*). Since some studies suggested that middle-aged people reacted more positively to CSR actions than younger or older people (e.g., Tian et al., 2011), *age squared* was also included in the analysis to detect possible non-linear effects.

### Data Analysis

The empirical analysis strategy consisted of three steps. First, descriptive statistics were provided to give information about the sample structure. Second, four ordinary least squares (OLS) regression analyses were carried out to investigate the determinants of CSR perception and answer the second research question. The dependent variables were the CSR measures for the full sample and for the DFL, UEFA, and IOC sub-samples. The remaining variables from Table 3 were included as independent variables. The first model also included dummy variables for the three sport organizations to check for organizational differences. To control for regional differences because of the different regulations enforced by state governments during the pandemic, the models included state dummies. Robustness checks were conducted with the CSR measure for the full sample as a dependent variable in three additional models (Models 5, 6, 8). Model 5 used a Tobit regression, Model 6 excluded the pandemic-related variables, and Model 8 included an interaction term between sport interest and gender. Model 7 was calculated with a weighted CSR scale, considering the strength of each item following the second-order CFA. All models were estimated with heteroscedasticity-consistent standard errors. The independent variables were checked for multicollinearity using correlation analyses (Table 6) and variance inflation factors (VIFs). Since *CC sport recent* and *CC sport lockdown* as well as *CC interest* and *CC frequency* were highly correlated, only *CC sport lockdown* and *CC frequency* were included in the regression models. Since all other correlation coefficients, except for age and its squared term, which are naturally correlated, were below 0.8 and all VIFs were under the critical value of 10, multicollinearity should not present a problem for the present analysis (Hair et al., 2010).

In a third step, a two-step cluster analysis with the CSR scale as a cluster variable was conducted to answer the third research question. Three different clusters resulted from this analysis. Analyses of variance were employed to detect differences between clusters with regard to population characteristics. In the case of significant ANOVA results, a Bonferroni *post-hoc* test was applied to identify between which clusters the differences were statistically significant.

## Results

Table 3 displays the descriptive statistics. Altogether, 49.3% of the respondents were male. The average age was 49.62 years, which was slightly higher than the German population average of 45 years^1^. Nearly half of the respondents had at least A-levels, whereas 45.3% had an educational degree below A-levels. The average personal monthly net income was between €2,000 and €2,500. Most respondents were full-time or part-time employed (60.4%), while pensioners represented the second biggest group with 27.2%.

Respondents showed moderate to strong general interest (*M* = 3.4) and involvement (*M* = 4.48) in sport. They participated 7.37 h in sport per week. This rather high value might be due to the sports context of the study because it was only representative regarding age, gender, and federal states. The interest in the competitions provided by the governing body and the frequency of watching them were highest for the IOC, followed by UEFA and DFL.

The summary statistics for perceived CSR showed that the activities of the DFL (*M* = 4.21) and UEFA (*M* = 4.23) were rated higher than the activities of the IOC (*M* = 4.05). Moreover, the CSR dimensions were perceived as significantly different from each other, with the economic and legal dimension being rated highest and the philanthropic dimension being rated the lowest.

Table 8 summarizes the results of the regression models. The models explained between 20.7 (full sample) and 28% (UEFA) of the variances in perceived CSR. Starting with the socio-demographic variables, gender was the only variable that was significant in all models. The negative coefficients showed that males evaluated the activities as significantly worse regarding their social responsibility than females. Age was only significant in the IOC model. Its negative effect, together with the positive effect of the squared term, indicated a U-shaped relationship between perceived CSR and age. The turning point for the CSR perception was at 45.13 years. This means that the evaluation of CSR activities decreased until the age of 45 and increased afterwards. In the full model, the coefficient for workers was positive and significant, meaning that full-time and part-time employees perceived the actions as more positive than the unemployed population. Furthermore, individuals with a migration background rated the CSR of sport organizations higher than individuals without a migration background.

**Table 8:** Regression models for perceived CSR.

	**Model 1:**	**Model 2:**	**Model 3:**	**Model 4:**
	**CSR**	**CSR DFL**	**CSR UEFA**	**CSR IOC**
Sport participation	0.001	−0.004	0.011	−0.003
Sport interest	−0.024	−0.130	0.056	0.076
Involvement	0.082^**^	0.242^***^	0.021	−0.042
CC sport lockdown	0.046	0.016	0.027	0.086
CC news recent	0.026	0.161	0.017	−0.066
CC news lockdown	0.033	−0.121	0.022	0.186^*^
CC frequency	0.287^***^	0.166	0.387^***^	0.289^**^
WHO-5	0.003	0.002	0.008^**^	0.000
Male	−0.482^***^	−0.475^**^	−0.329^*^	−0.654^***^
Age	−0.030	0.031	−0.017	−0.063^*^
Age squared	0.000	0.000	0.000	0.001^*^
A-levels	−0.060	0.075	−0.256	−0.135
University	−0.074	0.143	−0.172	−0.314
Income	−0.025	−0.012	−0.025	−0.022
Worker	0.312	0.325	0.206	0.491
Short-time work	−0.235	−0.171	−0.901	0.663
Student	0.224	0.598	0.284	0.380
Pensioner	0.069	0.226	0.012	−0.081
Migrant	0.228^*^	0.293	0.113	0.177
UEFA	−0.047	—	—	—
IOC	−0.208^*^	—	—	—
Federal state dummies	YES	YES	YES	YES
Constant	3.457^***^	2.179^*^	2.695^**^	3.793^***^
*R^2^*	0.207	0.276	0.280	0.252
*F*	6.966^***^	3.342^***^	3.407^***^	2.958^***^

* *p < 0.05*;

** *p < 0.01*;

*** *p < 0.001; displayed are the unstandardized coefficients; reference categories are low education, unemployed, German Bundesliga (DFL), and Bavaria*.

Consumption capital also affected perceived CSR, though mainly in the form of sport-specific consumption capital. Specifically, consumption frequency significantly influenced CSR perception in a positive manner. Those watching the competitions of the respective sport governing body more frequently evaluated its CSR activities more positively. Moreover, the full model showed a significant positive effect of involvement. Lastly, the IOC dummy was significantly negative in the full model, suggesting a lower CSR rating for the IOC than the DFL and confirming the tendencies of the descriptive statistics.

Table 9 provides the robustness tests for the conducted regression analyses, including a Tobit regression, interaction terms, and a weighted CSR scale. The conducted robustness tests showed strong robustness of the results, as the significances and direction of the effects remained similar in models 5–8.

**Table 9:** Robustness tests for regression models.

	**Model 5 (Tobit):**	**Model 6 (OLS):**	**Model 7 (OLS):**	**Model 8 (OLS):**
	**CSR**	**CSR**	**Weighted CSR**	**CSR**
Sport participation	0.001	0.000	0.000	0.001
Sport interest	−0.025	−0.013	−0.022	−0.056
Involvement	0.084^*^	0.095^**^	0.077^*^	0.082^**^
CC sport lockdown	0.044	—	0.039	0.046
CC news recent	0.027	—	0.025	0.027
CC news lockdown	0.035	—	0.033	0.033
CC frequency	0.296^***^	0.302^***^	0.274^***^	0.285
WHO-5	0.003	0.003	0.003	0.003
Male	−0.480^***^	−0.471^***^	−0.460^***^	−0.681^**^
Male x Sport interest			—	0.059
Age	−0.030	−0.028	−0.028	−0.031
Age squared	0.000	0.000	0.000	0.000
A-levels	−0.053	−0.052	−0.060	−0.057
University	−0.071	−0.060	−0.067	−0.073
Income	−0.029	−0.024	−0.025	−0.025
Worker	0.328	0.319	0.291	0.313
Short-time work	−0.244	−0.250	−0.234	−0.250
Student	0.246	0.280	0.218	0.218
Pensioner	0.045	0.055	0.024	0.035
Migrant	0.232	0.240^*^	0.213^*^	0.229
UEFA	−0.050	−0.037	−0.046	−0.043
IOC	−0.209^*^	−0.206^*^	−0.196^*^	−0.206^*^
Federal state dummies	YES	YES	YES	YES
Constant	3.402^***^	3.549^***^	3.271^***^	3.570^***^
*R^2^*	—	0.202	0.206	0.207
*Pseudo R^2^*	0.067	—	—	—
*F*	6.23^***^	7.432^***^	6.928^***^	6.793^***^

* *p < 0.05*;

** *p < 0.01*;

*** *p < 0.001; displayed are the unstandardized coefficients; reference categories are low education, unemployed, DFL, and Bavaria*.

The cluster analysis yielded three clusters, which are presented in Table 10. The three clusters were labeled “supporters” (23%), “neutral observers” (61.8%), and “critics” (15.2%). The mean value for the CSR perception decreased from 5.83 in cluster 1 to 1.93 in cluster 3. The *post-hoc* test confirmed significant differences in CSR perception among all three clusters. Moreover, the ANOVAs showed significant differences for all five CSR dimensions, with “supporters” reporting the highest mean values (e.g., 5.88 for the ethical dimension) and “critics” the lowest (e.g., 1.64 for the ethical dimension). Again, the *post-hoc* test confirmed that these differences were statistically significant between all three clusters.

**Table 10:** Results of cluster analysis and *post-hoc* test (displayed are the mean values).

	**Cluster 1:**	**Cluster 2:**	**Cluster 3:**	***F***
	**Supporters**	**Neutral observers**	**Critics**	
No. of obs.	230	618	152	
**Cluster variable**				
CSR scale	5.83^b^^c^	4.09^a^^c^	1.93^a^^b^	2381.75^***^
**CSR dimensions**				
Economic	5.89^b^^c^	4.32^a^^c^	2.53^a^^b^	789.17^***^
Philanthropic	5.56^b^^c^	3.87^a^^c^	1.67^a^^b^	1210.11^***^
Ethical	5.88^b^^c^	4.01^a^^c^	1.64^a^^b^	1581.46^***^
Legal	6.04^b^^c^	4.25^a^^c^	1.93^a^^b^	1269.11^***^
**Organizational characteristics**				
DFL	0.357	0.333	0.296	0.753
UEFA	0.344	0.337	0.303	0.389
IOC	0.300	0.330	0.401	2.17
**Individual characteristics**				
Sport participation	8.43	7.03	7.16	2.72
Sport interest	3.76^b^^c^	3.34^a^	3.13^a^	18.93^***^
Involvement	5.38^b^^c^	4.33^a^^c^	3.73^a^^b^	45.58^***^
CC sport lockdown	2.80^b^^c^	2.16^a^	2.02^a^	27.55^***^
CC news recent	3.74^b^^c^	3.44^a^	3.39^a^	6.04^**^
CC news lockdown	3.93^c^	3.74	3.64^a^	3.361^*^
CC frequency	3.67^b^^c^	2.96^a^^c^	2.46^a^^b^	51.59^***^
WHO-5	56.85	56.13	53.79	0.776
Male	0.48^c^	0.47	0.61^a^	5.18^**^
Age	48.84^c^	49.09^c^	52.91^a^^b^	3.83^*^
Low education	0.47	0.44	0.49	0.951
A-levels	0.26	0.26	0.19	1.73
University	0.27	0.30	0.32	0.564
Income	2304.35	2135.92	2246.71	2.21
Worker	0.67^c^	0.61^c^	0.49^a^^b^	5.76^**^
Short-time work	0.01	0.01	0.02	0.636
Student	0.06	0.05	0.04	0.311
Pensioner	0.23^c^	0.27	0.35^a^	3.50^*^
Unemployed	0.04	0.06	0.10	2.29
Migrant	0.20^b^	0.12^a^	0.14	4.26^*^

* *p < 0.05*;

** *p < 0.01*;

*** *p < 0.001; results of Bonferroni post hoc test*:

a *significantly different from cluster 1*;

b *significantly different from cluster 2*;

c *significantly different from cluster 3*.

The ANOVAs yielded no significant differences in organizational characteristics but in individual characteristics. The sports interest of “supporters” (3.76) was significantly higher than for “neutral observers” (3.34) and “critics” (3.13). However, the difference between cluster 2 and 3 was not significant. The clusters also differed in their level of involvement, with the *post-hoc* test suggesting that involvement was highest among the “supporters” (5.38), followed by “neutral observers” (4.33), and “critics” (3.73) indicating the lowest involvement. The sport-related consumption capital (CC sport lockdown; CC frequency) in particular differed between the three clusters, and the *post-hoc* test confirmed that the sport-related consumption capital of cluster 1 (2.8; 3.67) was significantly different from cluster 2 (2.16; 2.96) and 3 (2.02; 2.46). However, the *post-hoc* test also yielded significant differences between cluster 2 and 3 only for the consumption frequency.

For the socio-demographic characteristics, the ANOVAs and *post-hoc* tests yielded mostly significant differences between “supporters” and “critics.” “Critics” included significantly more males (61%) than “supporters” (48%). Furthermore, with 52.91 years on average, respondents from cluster 3 were significantly older than those in cluster 2 (*M* = 49.09) and cluster 1 (*M* = 48.84). The age difference between the first two clusters was not significant. “Supporters” and “critics” also significantly differed in the share of full-time employees and pensioners. While cluster 1 (67%) yielded a greater share of full-time employees than cluster 3 (49%), cluster 3 had a significantly higher share (35%) of pensioners than “supporters” (23%), which reflected the presented age differences.

## Discussion

The study set out to examine the CSR of three sport organizations during the first wave of the COVID-19 pandemic as perceived by the German resident population. The idea of focusing on the population's perception followed stakeholder theory, which points out the responsibility of organizations towards multiple stakeholders. In this case, society was the main stakeholder in this study, which was largely neglected in previous research. Furthermore, a positive CSR perception within the population could help legitimize the special role of sport organizations in the ongoing pandemic.

Corporate social responsibility was measured using an existing scale, which was adapted for this research context. The conducted CFA and correlation analysis showed the reliability and validity of the scale. This finding answered the first research question and showed that the scale was appropriate for the sport governing context.

Similar to the perception of member federations of the UEFA toward their own activities (Walters and Tacon, 2011), the CSR perception of the population was split between high and low emphasis on social responsibility to the society. Hence, the CSR scale values slightly over four reflected that the perception the population has of CSR activities is mixed. Even though the UEFA and IOC are non-profit organizations, they generate enormous revenues, which could lead to the economic dimensions being rated highest (Chelladurai, 2016). Economic responsibility was measured among others with cost control and long-term and sustained success, which showed that the population perceived that the organizations managed their economic resources well to gain long-term viability. Moreover, the item measuring profit maximization was deleted, indicating that the remaining four items measured economic responsibility beyond mere profit seeking. The close cooperation of the organizations with politics was emphasized from both ends during the first wave of the pandemic, which ensured legal obligations were met and probably resulted in the overall high perception of the legal dimension. Overall, the results provided a starting point for measuring the CSR perceptions of sport organizations, which can serve as a reference point for future quantitative assessments of CSR perceptions at different levels (e.g., professional sport teams, non-profit sport clubs).

Comparing the three organizations, the DFL and UEFA scored similarly and higher than the IOC, which could be a result of the late decision-making by IOC officials regarding the Tokyo 2020 Olympic Games. As highlighted by stakeholder theory, all three organizations perceived pressure from the population as an external stakeholder to act responsibly during a worldwide pandemic, with the earlier decisions by the DFL and UEFA seemingly being perceived positively regarding their societal responsibility.

To answer the second research question, regression analyses were conducted, while a cluster analysis was also performed to answer the third research question. In the regression results, gender was the only consistently significant socio-demographic variable revealing a more positive evaluation of CSR activities by women than by men. Previous CSR research (Patino et al., 2014; Kim and Kim, 2016) and studies on the socially conscious consumer (Gupta and Singh, 2017) also showed that women were more socially conscious and valued CSR activities. Even though it seems that women are more critical toward socially responsible behavior, they still perceived the activities of the three organizations as more positive. This finding was also reflected in the results of the cluster analysis. While the cluster “supporters” was gender-balanced, the “critics” were skewed toward males. Men perceived the activities as more critical, which may have been a result of the games being played without spectators, as more men than women enjoy the stadium atmosphere. However, the interaction term between sports interest and gender was insignificant, indicating that the more negative perception for men does not interact with their general interest in sports.

The inconsistent age effect of the regression models is similar to the results of Pérez and Rodríguez del Bosque (2013a). Previous studies showed that it is difficult to detect consistent age effects using regression analyses. However, a closer look at the cluster analysis revealed age differences between the clusters. While the “supporters” and “neutral observers” were relatively younger, the older composition of “critics” came along with a higher share of pensioners. Since the older and retired population was greatly threatened by the coronavirus, it may seem plausible that they opposed the actions of the three governing bodies, especially the continuation of competitions. In contrast to pensioners, full-time employees were better represented among the “supporters.” The three organizations constantly stated that sport is the profession of their members, thus insisting that they should be allowed to exercise their profession similar to other businesses. This was also the reason why the German government allowed the continuation of competitions at the professional level (German Government, 2021). This view might have strengthened the acceptance among employees, who themselves exercised their professions during the pandemic. One might argue that they were also the ones who did not lose their jobs; hence, they had a higher acceptance for the competitions to continue, but due to large short-time work programs in Germany, only a slight increase in the unemployment rate was observed in Germany and job losses were rare.

Sport-related consumption capital, involvement, and the overall interest in sports not only influenced CSR perception positively but were also significantly different between the three clusters. The generally strong interest and high weekly participation in the sample might have been due to the sports context of the survey, which might have led to self-selection of respondents from Toluna's panel. However, this phenomenon occurs frequently in sport surveys (e.g., Wicker and Hallmann, 2013; Lacey and Kennett-Hensel, 2016), and the sampling procedure tried to minimize this phenomenon by being representative regarding age, gender, and federal state. The consumption frequency of competitions turned out to be a more effective measure of sport-related consumption capital than following sport news. While sport events can be consumed weekly (German Bundesliga), monthly (UEFA competitions), or at least every two years (Olympic Games), sports news can be consumed every hour, which makes a more distinctive perception of news more sophisticated. Those who consumed the competitions and sports news more frequently were in the “supporters” cluster, while those with less frequent consumption were in the “neutral observers” and “critics” clusters. More frequent consumption also influenced CSR perception positively in the regression analyses. Therefore, repetitive consumption of sport increases both utility, as explained by consumption capital theory, and CSR perceptions. Overall, it appears that news consumption is less important for the forming of an opinion about activities than actively watching games or competitions. Additionally, the general interest in sports and levels of involvement were significantly higher for “supporters” than for the two other clusters. Those with high interest and high involvement are more likely to actively inform themselves about activities (Du et al., 2010) which might lead to a better perception regarding the societal responsibility of those actions.

These findings have practical implications for sport managers and politicians. Major sports organizations, such as the DFL, have a special role during the pandemic. Even though lockdowns and travel restrictions are in place, politicians allow professional sports leagues to continue their competitions. This might be legal because other businesses partially enjoyed exemptions from travel and contact restrictions as well. However, social responsibility includes behaviors beyond legal obligations, which means that not all behavior that is legal is also viewed as legitimate by the population (Carroll, 1991). It is a positive sign for sport managers to see that people with higher and repetitive consumption evaluate their activities more favorably. However, large parts of the population are undecided on how to value the societal responsibility of the actions during the first wave of the pandemic or value them low. To further justify such a special role during the pandemic, it is important that societally responsible actions are broadly communicated and that the organizations avoid irresponsible behavior. Otherwise, the legitimacy of the special role of sports will be doubted. Moreover, the elderly population in particular perceived such actions critically. Since this population group makes up a large part of the spectators, it is important for the organizations to adequately protect this group in case of the complete return of fans. Otherwise, the threat of infection might hinder them from returning into the stands. For politicians, the results of the cluster analyses in particular yielded interesting insights. The acceptance of the actions of the three sports governing bodies, i.e., the continuation of competitions, were especially low among the older population and pensioners, who were threatened the most by the virus. Since the elderly population makes up a large part of the electorate, and restrictions during the pandemic (e.g., lockdowns and curfews) can only be sustained with great solidarity and acceptance, the special role of sports should be thoroughly monitored.

## Conclusion

The study investigated the CSR of the DFL, UEFA, and IOC during the first wave of the COVID-19 pandemic as perceived by the German resident population. The lockdown and travel restrictions in March and April presented a situation that has never existed before for the German population and the world of sports. Since then, sport organizations have faced trade-offs between economic viability and social responsibility. Due to the special role that the politics attributed to professional sport leagues, competitions were allowed to restart in May. However, there was constant criticism from the population toward exceptions for players and athletes, especially from the segments of the population that were most threatened by the pandemic and that rarely consumed sport, who responded to their actions critically. Sport-governing bodies should be aware of such criticisms. They are well-advised to remain humble to maintain acceptance from their core stakeholders, and can thus be further granted exceptions and survive the economic threats conveyed by the pandemic.

The study makes several contributions to the literature. Through the lenses of stakeholder theory and consumption capital theory, the importance of sport consumption in explaining perceived CSR was shown for an often-forgotten stakeholder of CSR activities, i.e., society itself. For the first time, the socio-demographic factors of CSR perception were developed in sport research based on the concept of socially conscious consumers. Empirically, a starting point for future quantitative assessments of CSR activities was provided. The study also relied on a comprehensive and representative sample, subsequently reducing the possibility of biased perceptions from the oversampling of population groups, such as males or highly educated respondents, who are often overrepresented in other sport surveys.

The study comes with some limitations, which can guide future research. First, the generalization of the findings was limited in two ways. First, on one hand, the study only surveyed the German population. Hence, the findings regarding the perceptions of the populations were limited to the scope of Germany. On the other hand, the perceptions in this study represented the perception of the three sport organizations after the first wave of the pandemic. It might be interesting to compare the presented values with future perceptions after the pandemic and check if increasing or decreasing tension in society influences CSR perceptions, specifically with certain controversies regarding the 2021 UEFA European Championships and the 2021 Tokyo Olympic Games. Second, this study focused only on sports governing bodies with high revenues. It would be interesting to detect the differences and similarities in the perception of the activities of community sport clubs and if the same individual characteristics determine these perceptions and if the same clusters emerge based on these perceptions. Future studies among community sport clubs and professional sport teams could increase the comparability of the initial CSR values of this study. Third, the quantitative research design of this study did not allow for an in-depth understanding of the reasons for low CSR perceptions. Future research could use a mixed-methods approach to gather in-depth knowledge about the drivers of CSR evaluations by the population.

## Data Availability Statement

The raw data supporting the conclusions of this article will be made available by the authors, without undue reservation.

## Author Contributions

TT drafted the introduction, literature review, methods, results, discussion, and conclusion. PW advised the data cleaning and data analysis and handled the communication with Toluna Germany and also checked the overall manuscript for coherence, language, and format. TT and PW both qualify as authors and all those who qualify for authorship are listed. All authors were part of the research from the very beginning, designed the questionnaire, developed the conceptualization of the study, revised the manuscript critically for important intellectual content, and agreed on the final version of the manuscript.

## Conflict of Interest

The authors declare that the research was conducted in the absence of any commercial or financial relationships that could be construed as a potential conflict of interest.

## Publisher's Note

All claims expressed in this article are solely those of the authors and do not necessarily represent those of their affiliated organizations, or those of the publisher, the editors and the reviewers. Any product that may be evaluated in this article, or claim that may be made by its manufacturer, is not guaranteed or endorsed by the publisher.

## Footnotes

^1^This minor difference is rooted in the assessment of age using an open question in the survey, while the micro census uses age categories.
